# 

**Published:** 1902-09-06

**Authors:** 


### NOTICE TO CORRESPONDENTS.

All MSS., Letters, Books for Review, and other matters intended for the Editor should be addressed The Editor, "The Hospital," The Hospital Building, 28 & 29 Southampton Street, Strand, W.C.

The Editor cannot undertake to return rejected MS., even when accompanied by stamped directed envelope.

**Notice to Advertisers and Subscribers.**

All Advertisements, Orders for Copies of The Hospital, and Business Communications should be addressed to **THE MANAGER, (Not to the Editor)**, The Hospital, 28 & 29 Southampton Street, Strand, London, W.C.

The Cost of Subscription, post free, is as follows.—Per week, 2½d.; per quarter, 2s. 8d.; per half-year, 5s. 3d.; per annum, 10s. 6d. Foreign:—Per annum, 15s. 2d., payable, in all cases, in advance.

**NOTICE.—Vol. XXXI. of "The Hospital" (October 1901, to March 1902), in handsome cloth binding is now on sale at the office of this Journal, price, 7s. 6d. Cases for binding the Numbers contained in this Volume 1s. 6d. Index to Vol. XXXI., 2½d., post free.**

**The Monthly Part for September is now ready. Price 6d., by post 8d.**

### BOOKS RECEIVED.

Diprose, Bateman and Co.

"The Organisation of the Public Health Department in Dealing with the Epidemic of Small-pox in the Metropolitan Borough of Holborn." By Wilfred Watkins-Pitchford, M.B., F.R.C.S., D.P.H.

Baillière, Tindall and Cox.

"Notes on Medicine, for Medical and Dental Students." By W. D. Woodburn, L.D.S.

"Aids to Dental Anatomy and Pathology." By Arthur S. Underwood, M.R.C.S., L.D.S.

### SCRAPS AND GLEANINGS.

Against the increase in wages in the United States of 12 per cent. since 1896, the cost of the necessaries of life has increased 30 per cent. in the same time.

Plague mortality in India is increasing at the rate of 1,000 cases per week.

It is stated that with the Kaiser's permission, and by desire of King George of Greece, a high German medical staff officer will shortly proceed to Athens to take over the organisation of the Greek army medical service.

In consequence of the epidemic of cholera at Alexandria and Cairo, the prefects of the Maritime Department of France have been warned that the sanitary measures prescribed for vessels arriving from infected ports are vigorously adhered to.

At Davos Platz, with a view of preventing the spread of tuberculosis, the authorities send a special emissary to board every train coming to the town, and serve each traveller with a print of the rule as to the prohibition of expectoration, the reasons for it, and the penalties attached to its breach.

The Committee of the Hospital for Sick Children, of which the Duke of Fife is president, have made Mr. F. W. Speaight an Honorary Life Governor of the hospital in recognition of his "great and valuable services" in having designed the Imperial Coronation Bazaar by which a sum of nearly £25,000 was raised.

### THE STUDENT OF MEDICINE.

### APPOINTMENTS.

**Arthur Cyril Bird**, M.R.C.S.Eng., L.R.C.P.Lond., appointed Medical Officer to the Sidmouth Dispensary. **John D'Ewart**, M.R.C.S., L.R.C.P., appointed House Physician to the Manchester Royal Infirmary. **F. R. Rawdon**, M.B., Ch.B., appointed House Surgeon to the Rotherham Hospital and Dispensary. **Ward Smith**, M.B., Ch.B.Edin., F.R.C.S.Eng., appointed Surgeon to Sir Titus Salt's Hospital, Shipley. **W. A. Valentine**, M.D., B.Ch.Dub., appointed Honorary Surgeon to the Bideford and District Dispensary and Infirmary. **T. Walker**, M.R.C.S., L.R.C.P., appointed House Surgeon to the Manchester Royal Infirmary. **Arthur G. Wilkins**, M.B., Ch.B., appointed Resident Medical Officer to St. Mary's Hospital for Women and Children, Manchester.

### "The Hospital" Institutional Library.

"Hospitals and Asylums of the World." 4 Vols., with a Portfolio of Plans, complete £12 12s.

"Burdett's Hospitals and Charities, 1902." 5s.

"Cottage Hospitals, General, Fever, and Convalescent." 10s. 6d.

"Hospital Expenditure: The Commissariat." 2s. 6d.

"A Legal Handbook for the Use of Hospital Authorities." 2s. 6d.

"The Cottage Hospital Case Book or Register of Patients.' 10s. and 12s. 6d.

"The Furnishing and Appliances of a Cottage Hospital." 6d.

All these are published by the Scientific Press, Ltd., and may be obtained through any bookseller, or direct from the publishers, 28 and 29 Southampton Street, Strand, London, W.C.

